# Endoplasmic Reticulum Stress in Hepatitis B Virus and Hepatitis C Virus Infection

**DOI:** 10.3390/v14122630

**Published:** 2022-11-25

**Authors:** Tengyue Hu, Jiayi Wang, Weixiu Li, Miao Liu, Ning Han, Man Yuan, Lingyao Du, Hong Tang

**Affiliations:** 1 Center of Infectious Diseases, West China Hospital of Sichuan University, Chengdu 610041, China; 2 Division of Infectious Diseases, State Key Laboratory of Biotherapy and Center of Infectious Diseases, West China Hospital, Sichuan University, Chengdu 610041, China

**Keywords:** hepatitis B virus, hepatitis C virus, endoplasmic reticulum stress, unfolded protein response

## Abstract

Endoplasmic reticulum (ER) stress, a type of cellular stress, always occurs when unfolded or misfolded proteins accumulating in the ER exceed the protein folding capacity. Because of the demand for rapid viral protein synthesis after viral infection, viral infections become a risk factor for ER stress. The hepatocyte is a cell with large and well-developed ER, and hepatitis virus infection is widespread in the population, indicating the interaction between hepatitis viruses and ER stress may have significance for managing liver diseases. In this paper, we review the process that is initiated by the hepatocyte through ER stress against HBV and HCV infection and explain how this information can be helpful in the treatment of HBV/HCV-related diseases.

## 1. Introduction

Viral hepatitis is a serious public health problem affecting hundreds of millions of people worldwide, which results from hepatotropic virus infection, including hepatitis virus A–E. It is estimated that, in 2015, 257 million people were living with hepatitis B virus (HBV), while 71 million people were living with hepatitis C virus (HCV), globally [1]. HBV and HCV are the two major viruses that could cause a persistent infection status of the liver, also called chronic infection. Chronic HBV and HCV infections are associated with hepatic fibrosis and hepatocellular carcinoma (HCC) [2,3]. Although antiviral therapies against HBV and HCV have been greatly developed, antiviral resistance is still a major obstacle for anti-HBV treatment and may become a potential obstacle for anti-HCV treatment in the future, prompting researchers to explore novel therapeutic targets [4]. Endoplasmic reticulum (ER) stress, a type of cellular stress, is reported to play an important role in the development and treatment of chronic HBV and HCV infection, as well as related liver diseases.

ER, a vital organelle, plays a major role in the synthesis of proteins, membrane proteins, and lipids, and is particularly important in the folding and structural maturation of secretory proteins [5,6]. Cells always work at full secretory capacity, resulting in excessive protein folding stress for ER [6]. ER stress always occurs when unfolded or misfolded proteins accumulating in the ER exceed the protein folding capacity [6,7,8]. Hepatocytes have a well-developed ER, which enables them to secrete high levels of proteins efficiently [6,9]. However, virus infection status exerts a great deal of stress on the ER, making ER stress a more common situation in chronic hepatitis. This article reviews the process that is initiated by the hepatocyte through ER stress against HBV and HCV infection and explains how this information can be helpful in the treatment of HBV/HCV-related diseases.

## 2. ER Stress in the Liver

### 2.1. Risk Factors for ER Stress

ER stress is a state in which the ER is under stress. It appears when unfolded or misfolded proteins accumulating in the ER lumen exceed the protein folding capacity, as well as too many other substances accumulating in the ER [6,7,8]. Risk factors that can cause ER stress reported by previous studies include elevated protein synthesis, accumulation of unfolded proteins, lipid, and nucleic acid in the ER, glucose deprivation and altered glycosylation, ER calcium (Ca^2+^) depletion, as well as the alteration of redox status [10,11,12]. Because of the highly active synthesis of proteins and lipids in the liver, conditions that can change protein folding or lipid metabolism are able to lead to ER stress. It has been reported that viral infections, alcoholic liver injury, non-alcoholic fatty liver disease (NAFLD), diabetes and diabesity, and HCC are related to the occurrence of ER stress, which can in turn accelerate the progress of these conditions [13,14,15,16,17]. In our study, we focus on the association between HBV, HCV and ER stress in the liver.

### 2.2. The ER Stress Response and Related Mechanisms

Cellular homeostasis will be disrupted when high-intensity and prolonged ER stress exists [18]. To restore homeostasis and avoid cell death, cells respond to ER stress through complex mechanisms, known as the ER stress response. The unfolded protein reaction (UPR) is the most widely studied mechanism in the ER stress response. In addition to the pathways of the UPR, many other factors are also involved in ER stress, including reactive oxygen species (ROS), Ca^2+^ and the sterol regulatory element binding protein (SREBP) [19,20].

ER stress response elements (ERSEs) are a group of short DNA sequences located within a gene promotor region that contains binding sites for specific transcription factors which regulate ER stress, and were first described 20 years ago [21]. ERSE-I (CCAAT-N_9_-CCACG) was shown to be necessary in inducing GRP78 and GRP94 transcription by analyzing the promoter regions of GRP78, GRP94, and calreticulin genes [22]. ERSE-II (ATTGG-N-CCACG) was found in the promotor region of human *Herp*, which encodes a 54-kDa membrane protein in the ER [23]. Unfolded protein response element (UPRE) was another element containing the consensus sequence TGACGTGG/A, and was one of the cis-acting elements capable of binding to the molecules in the pathways of the ER stress response [24]. The related pathways of the ER stress response will be described in the following sections.

#### 2.2.1. UPR in the Liver

**Figure 1:**
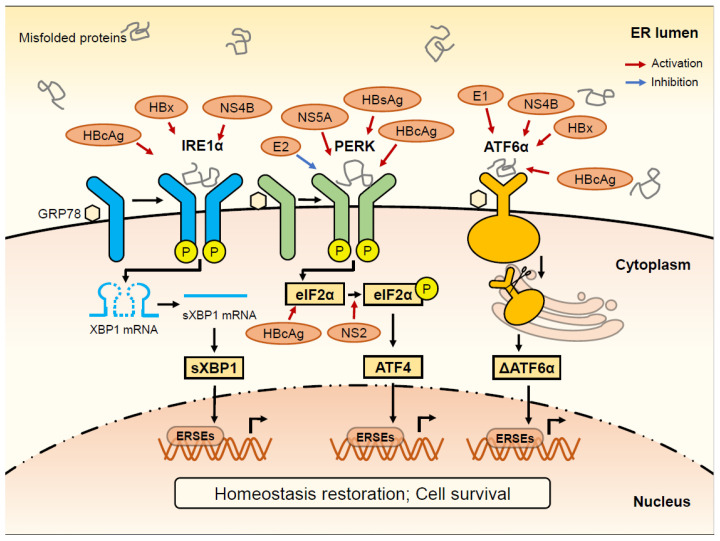
Mainstay of the three signal transduction pathways of the unfolded protein reaction (UPR). IRE1α, PERK and ATF6 are transmembrane proteins localized on the endoplasmic reticulum (ER) membrane and can be activated by the misfolded proteins in the ER lumen. In the cytoplasm, a 26-nt intron in X-box-binding protein 1 (XBP1) mRNA can be excised by the IRE1α, and then converted to its mature form encoding spliced XBP1 (sXBP1). PERK could phosphorylate eIF2α, and therefore promote the ATF4 expression. ATF6α translocates to the Golgi apparatus where it is cleaved to form cleaved ATF6α (ΔATF6α). sXBP1, ATF4, and ΔATF6α translocate to the nucleus where they are able to regulate the transcription of ER stress-responsive genes. ΔATF6α, sXBP1, and ATF4 also interact with several ER stress-response elements (ERSEs), resulting in the transactivation of unfolded protein response target genes, and finally function to restore homeostasis and avoid cell death.

UPR, the main component of the ER stress response, can be induced by the excessive ER stress to correct the condition where the misfolded and unfolded proteins in the ER exceed above a threshold [18,25,26,27,28]. The mechanisms initiating UPR in the liver did not demonstrate a distinctive pattern based on the existing literature. Three signal transduction pathways (Figure 1) that can initiate UPR are mediated by inositol-requiring enzyme 1α (IRE1α), pancreatic endoplasmic reticulum kinase (PERK) or activating transcription factor (ATF) 6, respectively [6,29]. GRP78, also known as BiP, binds and deactivates the three arms of the UPR in physiological status [30,31].

IRE1 signaling is the most widely studied pathways among the three pathways. IRE1 harbors an N-terminal luminal domain that can sense the protein-folding status within the ER. IRE1 possesses both Ser/Thr protein kinase and endoribonuclease (RNase) activities in its C-terminal cytoplasmic effector domain. IRE1α and IRE1β are two IRE1 isoforms encoded by mammalian genome, with IRE1α found to be the most abundantly and ubiquitously expressed [32]. IRE1α-X-box-binding protein 1 (XBP1) pathway is the most conserved branch of the UPR pathways. After the luminal N-terminal domain binding to misfolded proteins, IRE1α dissociates from the GRP78 binding complex and then its kinase becomes activated to trans-autophosphorylate Ser/Thr residues on the cytosolic tail [6,29,33]. Afterwards, the adjacent RNase is activated, which is essential for the UPR [29,34]. A 26-nt intron in XBP1 mRNA can be excised by the RNase, and then converted to its mature form encoding spliced XBP1 (sXBP1) [35]. The sXBP1 is a homeostatic transcription factor with the ability to induce transcription of many genes modulating the ER function after translocating to the nucleus [36]. However, conditions for the IRE1α-XBP1 pathway activation remain unclear. Some researchers believed that only high levels of ER stress would initiate the IRE1α-XBP1 pathway, without further descriptions on the level [6]. Moreover, the IRE1α-c-Jun N-terminal kinase (JNK) pathway is another pathway that is also reported in the liver [37,38]. The IRE1α-JNK pathway is more complex than the IRE1α-XBP1 pathway and JNK protein kinases can be activated by IRE1α upon ER stress [39]. However, how it operates in the liver requires verification. It has been accepted that apoptosis signal-regulating kinase 1 (ASK1) is the upstream signaling molecule of JNK and downstream molecule of IRE1α [40,41,42,43]. This pathway can lead to cell death in the liver [41]. The downstream molecules of JNK are diverse among studies, with the C/EBP homologous protein (CHOP), beclin-1, caspase-3 being reported [44,45,46].

PERK exhibits protein kinase activity upon ER stress, which could phosphorylate eukaryotic translation initiation factor 2α (eIF2α), and then gives the cell more time to fold proteins in the ER lumen by slowing protein translation [6,29,47]. When the amount of unphosphorylated eIF2α decreases to a certain level, translation of ATF4 will be upregulated [6]. It has been observed in the liver that CHOP was upregulated after ATF4 expression, frequently resulting in hepatocyte apoptosis [48,49,50]. Expression of dual-specificity phosphatase 5 (DUSP5) was reported to be increased via the PERK-CHOP pathway, leading to hepatocyte death [51]. Other details of PERK-related pathways in the liver remain unclear.

ATF6 has two subtypes, ATF6α and ATF6β, with ATF6α being reported more frequently in ER stress [52]. After the appearance of misfolded proteins, ATF6α translocates to the Golgi apparatus where it is cleaved by the Site-1 and Site-2 to release a cytosolic fragment (ΔATF6α). ΔATF6α translocates to the nucleus to regulate the transcription of ER stress-responsive genes, and therefore enlarges the ER lumen and increases ER folding capacity. Evidence also demonstrated that ΔATF6α could induce XBP1 transcription [53].

#### 2.2.2. Other ER Stress Response Pathways in the Liver

In addition to the pathways of the UPR, the ER overload response (EOR) is also a typical ER stress response. Different from the UPR, the EOR pathway releases Ca^2+^ from ER lumen to induce ROS production, and then activates nuclear factor-kappa B (NF-κB) [19,54]. The initiation of the EOR pathway is still unclear, and one study showed that the expression of the NS4B protein of HCV activated the pathway [19]. In fact, crosstalk of ROS and NF-κB signaling is complex, which has been studied intensively [55].

Studies also reported other molecules such as SREBPs and chaperones, activated in response to ER stress. However, their actions were not named as the ER stress response and were not verified in HBV-infected or HCV-infected cells [20,56,57]. As a result, when researchers mention the ER stress response, they refer to the UPR.

## 3. Viruses and the ER Stress Response

The ER participates in all stages of the virus replication cycle of HBV and HCV [30,58]. A large number of viral proteins and replication intermediates accumulate during viral replication, directly inducing ER stress [59]. Additionally, the ER will enter a stress state when there is an imbalance between the demand for rapid viral protein processing and the capacity of normal protein processing [28]. The ER stress response, represented by the UPR, will be induced by the infected cells to restrict viral activities. However, viruses have evolved some mechanisms to overcome this inhibition of viral replication [11]. A recent study reported that HBV can promote the viral replication by enhancing use of the autophagosome/multivesicular body axis after ER stress [60]. Here, the interactions between viruses and the pathways of the ER stress response will be described.

### 3.1. Viruses and the IRE1α Arm of the UPR

The researchers found that in HCV-infected cells, the expression of XBP1 is elevated, but the trans-activating activity of XBP1 is repressed, preventing the transcriptional induction of ER degradation-enhancing-mannosidase-like protein (EDEM). As a result, misfolded proteins are stable in these cells, and then the initiation of the URP pathways is inhibited [61]. It has also been observed in cytomegalovirus (CMV)-infected cells that the IRE1-dependent splicing of XBP1 was triggered, but the gene target EDEM did not accumulate [62]. The researchers believed that in this way, HCV may suppress the IRE1α-XBP1 pathway and then persist in infected hepatocytes [61]. However, the IRE1α-XBP1 pathway was considered to be activated by HBV [31]. HBV X protein (HBx) was considered as an adaptor or kinase activator to enhance the phosphorylation of IRE1α [63]. After the activation of the IRE1α-XBP1 pathway, the expression of EDEM was enhanced to limit the total amount of protein and reduce stress, to protect HBV-infected cells. Through the opposite mechanism, HBV also achieved persistence and chronic inflammation in hepatocytes [8].

### 3.2. Viruses and the PERK Arm of the UPR

The activation of the PERK pathway can repair the ER function, which is beneficial to viral replication. However, the PERK pathway also inhibits protein synthesis, which is unfavorable for viral replication and maturation. To maintain high-speed production of viral proteins, viruses must overcome the translation inhibition from the PERK pathway [58]. The E2 protein of HCV has been reported to bind to PERK as a pseudosubstrate, therefore sequester PERK from eIF2α. As a result, phosphorylation of PERK was inhibited, and HCV translation was enhanced. This mechanism also contributed to a persistent HCV infection [64]. In cells infected with herpes simplex virus (HSV), the oligomerization of PERK was observed when viral proteins were produced and processed in the ER [65]. Then, an increase in autophosphorylation of PERK was also observed, indicating PERK activation. The mechanisms by which HBV activates the UPR have not been fully clarified. A recent study showed that the PERK pathway can be induced by overexpression of hepatitis B surface antigen (HBsAg) [66]. In this study, signs of apoptosis were also observed upon prolonged activation of the PERK pathway.

In fact, the PERK-eIF2α-ATF4 pathway is considered to be the most important arm of the URP to regulate autophagy [8,67,68]. CHOP is an important transcription factor mediating and inducing ER stress-induced apoptosis [18,69,70]. The viral infections including hepatitis virus infection are reported to activate the three arms of the UPR, thereby inducing the CHOP expression, while the eIF2α is the most widely reported upstream molecule [71,72,73,74]. In HCV-infected cells, some researchers found that the CHOP expression did not increase, and the other researchers found that it increased after degradation of HCV core and NS5A protein [67,75]. HBV was reported to inhibit CHOP expression through the PERK pathway in HCC cells [76,77].

### 3.3. Viruses and the ATF6 Arm of the UPR

The ATF6 arm is the relatively simple one in the three arms. Various viruses were reported to induce the ATF6 pathway, including HBV, HCV, CMV, Epstein–Barr virus (EBV), African swine fever virus (ASFV), Rotavirus, Zika virus (ZIKA) and so on [58,63,78,79,80,81,82,83]. In HBV-infected cells, expression of HBx can activate the ATF6 pathway [63]. In HCV-infected cells, accumulation of unfolded major histocompatibility complex (MHC) class I may contribute to the activation of ATF6 [84].

Many viruses, including HCV, tend to only activate the ATF6 arm, with no upregulation of target chaperone proteins [79,80]. Some researchers thought that the induction of PERK and IRE1α arms may be detrimental for the viruses and the host cell, but the induction of the ATF6 arm was beneficial for virus replication and cell viability [58,79]. The mechanism and significance of the selective activation remain unclear. However, the activation of the three UPR arms have all been observed in HBV-infected cells, although the mechanism and significance also need further studies.

## 4. HBV and ER Stress

HBV is a small enveloped DNA virus in the *Hepadnaviridae family* [9]. HBV genome is a partially double-stranded circular DNA molecule including four overlapping open reading frames (ORFs) encoding core protein (pre-C, C), surface protein (pre-S1, pre-S2, and S), DNA polymerase (HBV Pol), and HBx [85]. A large number of proteins accumulate in the endoplasmic reticulum, resulting in ER stress, which may further aggravate liver diseases, leading to liver cirrhosis and HCC [60]. Here, we clarify ER stress caused by various HBV protein components and the effects on the occurrence and development of HCC after HBV infection, hoping to provide a new perspective for HBV treatment. HBV and associated ER stress pathways are shown in Table 1.

### 4.1. HBsAg and ER Stress

HBsAg can be divided into three types: hepatitis B virus large-surface proteins (LHBs), hepatitis B virus middle-surface proteins (MHBs), and hepatitis B virus small-surface proteins (SHBs). The LHB is composed of Pre-S1, Pre-S2 and S domains, while the MHB contains Pre-S2 and S domain. Additionally, the SHB is made up by S domain only [92]. Any of the sub-protein expression genes mutated would lead to the accumulation of surface proteins in the ER, resulting in ER stress.

HBV carrying natural mutations in the pre-S or S region is frequent, of which pre-S deleted mutation is even more common. The pre-S deletion mutated HBsAg can accumulate to a significant amount in the ER, which can initiate ER stress [66,93,94,95]. It has been observed in ground glass hepatocytes (GGH) that both types of pre-S mutants, one with either pre-S1 or pre-S deletions and the other consistently with pre-S2 deletion, can induce ER stress. Studies have reported that deletion in pre-S1 can activate GRp78, as well as upregulate PERK and c-JNK, which contribute to the increase in HCC-related factors, including cyclooxygenase (COX)-2, Cyclin A, NF-κB, and ROS [96,97,98,99,100,101]. Compared to pre-S1, the level of ER stress signals induced by pre-S2 mutants is lower [86]. Pre-S2 mutation can lead to the over-expression of MHBs, which would partially induce ER stress, activating ER stress-associated proteins such as IRE1, AFT4, CHOP, PERK, c-JNK, and sXBP-1 [96]. During ER stress, the production of IL-6 can be enhanced by the MHB via mitogen-activated protein kinase (MAPK)/NF-κB pathways [102]. It has also been demonstrated that point mutation of pre-S2 can activate GRP78 and GRP94, thus promoting the development of liver cancer. The S mutations include W36L, T47K, N52D, V184A, F220L, existing in the ER [99,101,103]. These molecules can up-regulate the expression of IRE1, ATF6, PERK, eIF2, XBP1, CHOP, and GRP78, particularly increasing hepatocyte apoptosis [104]. Although intracellular retention of HBsAg inducing ER stress is reported by previous studies, the mechanisms are still uncertain. A recent study showed that apolipoprotein H (APOH) can drive the retention of HBsAg, but did not explain why pre-S deficient mutants tend to be accumulated in the ER [105]. Further studies are needed to explore the detailed mechanism by which the mutation affects the accumulation of surface proteins in the ER.

Pre-S deleted mutants of HBsAg have been considered as important biomarkers for higher incidence rates of HCC development and potential targets in the diagnosis and treatment of HCC [92,94,95,106,107,108,109]. From a pathological point of view, previously mentioned GGH is the precancerous phenotype of hepatocytes, the appearance of which can be caused by HBV envelope proteins including HBsAg through the UPR pathways [66,90,92]. Through the PERK pathway in vitro, a study showed overexpression of HBsAg may be linked to the appearance of GGH [66]. In addition to histological observations, it was reported that the HBV pre-S gene deletions in the patient’s blood was related to liver disease progression, such as liver fibrosis and liver cancer and is associated with high risk of recurrence after radical resection of liver cancer [86]. Therefore, HBV pre-S gene deletions may be an indicator for evaluating disease progression and more attention should be paid to these patients with pre-S gene deletions. The ability to induce oxidative DNA damage might be a reason for the association between the pre-S deleted mutants and HBV-related HCC [86,95]. The small envelope protein of HBV was also a potential target for preventing and treating HCC, because of its ability to upregulate the expression of the vascular endothelial growth factor by activating the three arms of the UPR [110]. In summary, the relationship between ER stress, HBV, and HCC is extremely complicated and pathways of the UPR may play an important role in it.

The mechanisms for HBV-induced liver injury have been studied for several years. ER stress also participates in the liver injury. HBV is a type of the interferon (IFN) inducer. IFN exerts cytotoxicity to hepatocytes with HBsAg accumulating by suppressing the UPR, resulting in cell death [87].

### 4.2. HBx and ER Stress

HBx is an important factor in the developments of the HBV-induced diseases and a multifunctional regulator for ER stress [11,88]. Different from HBsAg accumulation which directly induces ER stress, HBx induces ER stress by reducing the cellular adenosine triphosphate (ATP) level, inducing stromal cell-derived factor-1 (SDF-1) over-expression and increasing hepatic lipid [11,88]. The chemokine SDF-1 is expressed in the healthy liver, but its expression increases when acute or chronic liver injury occurs. SDF-1 can promote liver fibrosis by recruiting mesenchymal cells from bone marrow. Furthermore, through enhancing the angiogenesis, tumor growth and metastasis, SDF-1 is involved in HCC facilitation [111]. In the process, ER stress response pathways interconnect with pathways in the inflammatory response, lipid metabolism and other unclear external pathways. The intracellular ATP levels were reduced by metabolic dysfunction. Subsequently, the PERK, eIF2α, and ATF4 pathways were activated, increasing COX2 mRNA and protein expression levels. Studies have also demonstrated that HBx induces ER stress through the IRE1-XBP1 and ATF6 pathways to improve hepatocyte survival [63]. In HCC cells, HBx is localized in ER lumen, interacting with GRP78 directly to inhibit eIF2α phosphorylation and suppress expression of ATF4 and CHOP, and then prevent apoptosis in HCC cells [112]. HBx was observed to interfere with the PERK pathway, which contributes to a reduction in eIF2α phosphorylation and ATF4, CHOP, and Bcl-2 expression. On the other hand, IRE1α, which generates pro-survival signals, was highly activated by HBx, and consequently, the expression of sXBP1 was increased [112]. In addition, the transcription of ATF3 and ATF6 has also been reported to be modified by HBx, resulting in the deregulation of the ER stress response [113]. In summary, HBx can regulate ER stress from multiple pathways, thereby preventing hepatocyte apoptosis. However, researchers also found that HBx induced apoptosis of hepatocytes by targeting protein phosphatase 2A-B56γ [114].

HBx is considered as the most oncogenic among the proteins encoded by HBV because of its ability to inhibit ER stress [113]. There are many studies which focus on how HBx initiates hepatocarcinogenesis. However, the mechanisms of HBx-induced HCC through regulating ER stress remain unclear. In a recent study, HBx showed its ability to enhance the development of liver fibrosis, still not indicating the association with ER stress [115].

COX2, an important mediator of inflammation, plays a role in crosstalk between inflammatory mediators and ER stress [116]. In addition, it has been reported that COX2 is co-expressed with HBx in the liver tissue from patients with chronic hepatitis [117]. HBx can induce the expression of COX2 through the eIF2α-ATF4 pathway, with the COX2 promoter playing a critical role [11].

### 4.3. HBcAg and ER Stress

Hepatitis B core antigen (HBcAg) is a single polypeptide which plays an important role in HBV infection. It can reflect the presence of Dane particles in serum and the replication of HBV in the liver, and can cooperate and complement each other with other HBV serological markers. Studies have shown that the mutation of HBcAg increases extracellular HBsAg. By activating the ATF6, GRP78, PERK, eIF2α, and IRE1α, HBcAg can increase inflammatory cytokines, ROS, cytochrome c, NF-κB, and TGF-β to regulate ER stress [91].

### 4.4. Other Factors Related to HBV and ER Stress

As one of the major organs for the metabolism of fat, the liver is susceptible to injuries by excessive accumulation of fat, so the signal pathways of lipid metabolism should be considered in the mechanism of liver injury [118]. Moreover, excessive intracellular lipid accumulation also induces ER stress with hepatocellular steatosis occurring simultaneously [119,120]. Other than HCV, there is little evidence that HBV affects lipid metabolism, but is has been proven that hepatocyte steatosis can induce ER stress to inhibit secretion of HBsAg and HBV DNA [121]. HBx can induce hepatic steatosis by activating lipogenic genes and down-regulating intracellular ATP levels, where researchers might explore a potential link between HBV, ER stress and lipid metabolism [122,123].

ER stress can be induced by alcohol in HBV-infected liver cells. There are a large number of studies on how alcohol induces ER stress in liver cells, but few studies have considered HBV together. A recent study showed that ER stress induced by alcohol suppressed HBV-peptide-MHC I complexes presented on hepatocyte surfaces, resulting in persistent HBV infection [124].

## 5. HCV and ER Stress

HCV is a small, enveloped RNA virus in the *Flaviviridae family*. The positive polarity RNA genome contained in HCV includes 5′ and 3′ untranslated regions (UTR) and a long ORF encoding a polyprotein precursor. At least 10 structural and nonstructural viral proteins have been reported to be processed by the polyprotein precursor, including core, E1, E2, p7, NS2, NS3, NS4A, NS4B, NS5A and NS5B [125]. Although the previous researchers did not observe apparent induction of UPR-responsive gene in livers from patients with untreated chronic hepatitis C, ER stress and activation of the three UPR pathways were observed [126]. HCV and associated ER stress pathways are shown in Table 2.

### 5.1. HCV Structural Proteins and ER Stress

Different from HBV, there are no detailed descriptions for one specific type of HCV protein in the previous studies in terms of their relation to ER stress. It has been reported that the HCV core protein induced ER stress by triggering overexpression of GRP78, GRP94, calreticulin and ATPase [128]. Later, in the yeast, the researchers found the immature core (aa 1–191, Core191) protein of HCV can induce UPR by inhibiting both ER-associated degradation (ERAD)-L, a degradation system whose main function is to maintain misfolded/unfolded proteins in the ER lumen, and ERAD-M, a degradation system mainly for proteins transporting a misfolded/unfolded region in the ER membrane, but the mature core (aa 1–177, Core177) protein, which only inhibits ERAD-L, was not able to induce UPR [127,128]. The degradation systems responsible for misfolded or unfolded proteins in the ER were also inhibited in the study, which was considered to be the reason for the UPR activation induced by the HCV core protein [127]. The expression of the NS2 protein can increase eIF2α phosphorylation to affect the PERK pathway, and ER stress inducible genes including GRP78 and ATF6 can also be upregulated by the NS2 protein [129]. The NS3 and NS4A proteins are reported to be inducers for ER stress, but mechanisms are not yet elucidated [129,130]. The NS4B protein can activate the IRE1 pathway by splicing XBP-1 mRNA, and then promotes HCV viral replication [131]. ATF6 was also considered to be used by the NS4B protein to induce the URP, but the mechanism is unclear [131]. The ATF4 and the ATF6 pathways induced by HCV structural proteins were reported to contribute to the induction of pro-apoptotic gene gadd153 [136].

### 5.2. HCV-Related Non-Structural Proteins and ER Stress

Although some researchers believed that the pathogenesis of persistent HCV infection resulted from ER stress and the UPR induced by HCV proteins, there is also relevant evidence of other molecules working during infection [127]. IFN-alpha receptor 1 (IFNAR1), a type of IFN receptor, can be downregulated by HCV through the PERK arm of the UPR in vitro. Knockout of PERK mitigates the degradation of IFNAR1, recovering the cell response to IFN and resistance to viruses [59]. Peroxisome proliferator-activated receptor gamma coactivator-1 alpha (PGC-1α) is a protein-regulating metabolism and inflammation by activating nuclear receptors [137]. The level of PGC-1α increases during ER stress caused by HCV, then enhancing HCV replication through phosphorylation of cyclic AMP (cAMP)-responsive element-binding protein (CREB) [59]. The protein kinase R (PKR), an innate immune pattern recognition receptor, is activated by double-stranded RNA (dsRNA) produced during HCV replication. The eIF2α can be phosphorylated by activated PKR to induce the UPR through the PERK arm [11,138,139,140]. Signal-peptide peptidase (SPP), an intramembrane protease, is involved in producing the mature core protein of HCV [141]. A 3:8 chromosomal translocation in hereditary renal cancer (TRC8) is a ubiquitin ligase participating in degrading the immature HCV core protein [141]. It was reported that HCV core protein expression altered ER distribution and induced ER stress in SPP/TRC8 double-knockout cells [141]. As a type of RNA virus, many biological processes of HCV are affected by the RNA modification N6-methyladenosine (m^6^A) [142]. The researchers found that the infection of HCV altered m^6^A modification of specific transcripts, including RIOK3 and CIRBP, and the changes in m^6^A in RIOK3 and CIRBP were partly caused by HCV-induced ER stress [142]. 

### 5.3. HCV-Related Lipid Metabolism Changes and ER Stress

It is widely accepted by researchers that infection of HCV leads to abnormal lipid metabolism in some patients, causing hepatic injuries such as non-alcoholic fatty liver disease [119,143]. Crosstalk between lipid metabolism pathways and the UPR pathways has been reported. JNK, a component of the IRE1α pathway with the capability to regulate lipid metabolism, contributes to intracellular lipid accumulation in the liver [144]. The phosphorylation of JNK aggravates HCV-infected hepatocytes lipoapoptosis [144]. In the pathogenesis of non-alcoholic fatty liver disease, XBP1 showed its ability to enhance both protein folding and lipid synthesis [33]. Whether XBP1 is an intersection of lipid metabolism pathways and UPR pathways in HCV infection still needs further investigation. 

SREBP is an important molecule to control cellular lipid metabolism [11,20,120]. The NS5A protein of HCV has been reported to induce hepatic lipid accumulation via the adenosine monophosphate-activated protein kinase (AMPK)-SREBP-1c pathway [132]. The links between ER stress and SREBP activation have also been reported in other conditions. Several research studies have reported that ER stress can dysregulate lipid metabolism through SREBP activating intracellular cholesterol concentration, and the functional peroxisomes are reported to play a role in preventing ER stress and imbalance of the sterol response pathway [132]. A recent study also reported that PERK was bound by SREBP to regulate ER stress and other cellular activities through the PERK signaling pathway [145]. As the existence of the links between HCV and abnormal lipid metabolism are generally accepted by researchers, it is necessary to verify the roles of SREBPs in the UPR in liver cells infected by HCV. Site-1 protease (S1P) and Site-2 protease (S2P) were observed more than 20 years ago to be shared by ATF6 and SREBPs in the Golgi apparatus, which was not verified in liver cells infected by HCV [146].

## 6. Conclusions and Perspectives

In our review, we tried to summarize how HBV and HCV performed in ER stress. Although the mechanisms are not clearly defined, it is clear that pathogenic processes after HBV or HCV infections have tight relationships with ER stress. Viral proteins are still pointcuts, of which the mechanisms are relatively clear in HBV and require more evidence for HCV. Another entry point, lipid metabolism pathway, has been proven to be affected by HBV and HCV activities. However, the role of ER stress in the interaction between lipid metabolism and HBV and HCV, especially HBV, requires further investigation. Other reviews also investigated ER stress and HBV and HCV. Some researchers investigated ER stress in common types of liver diseases including viral hepatitis, liver cancer, liver fibrosis and liver injury at the same time [147]. Virus infection was one of the causes of liver injury, and some researchers focused on liver injury and ER stress [10,148,149]. The relationship between hepatocarcinogenesis and ER stress was also a common topic [92,101,150]. Some researchers investigated HBV or HCV and ER stress [92,151,152]. In these previous reviews, these two hepatitis viruses and their associated ER stress pathways were not fully summarized. In our reviews, we focused on HBV and HCV to better explore therapeutic strategies for HBV- or HCV-related diseases.

Liver diseases are often accompanied by ER stress which can modulate infection, inflammation, autophagy and apoptosis, and cause liver injury. Paying attention to ER stress has great significance in the treatment of liver diseases including viral hepatitis [8]. The aim of studying the above mechanisms is to find better therapeutic strategies for HBV- or HCV-related diseases. Resistance against antiviral agents is still the major consideration in the development of new drugs, regardless of great advances in the area of antiviral treatment for hepatitis viruses [153]. For HBV infection, ER stress affects widely used treatment regimens, but the specific mechanism was not reported [154]. After entecavir treatment, clearance of extracellular HBV DNA can be accelerated, but clearance of intracellular HBV DNA may be delayed, which is thought to be caused by ER stress [154]. In the treatment for HCV infection, the selective pressure, an innate ability of viruses to evade antiviral therapy, always helps viruses successfully escape from direct-acting antivirals (DAAs) [155]. Studies on how ER stress works in resistance to antiviral therapy of hepatitis viruses are still lacking. In an in vitro experiment, ER stress was utilized by HCV to downregulate the expression of IFNAR1, influencing the antiviral effects of IFN-α [156]. Protein phosphatase 2A (PP2A), of which the upregulation is a result of ER stress, participated in the process [156]. 

There have been studies exploring potential therapeutic targets for anti-HBV and anti-HCV treatments. A specific treatment option has been proposed by one study [157]. Linking the expression of therapeutic genes to the UPR is thought to be a new option for anti-HBV therapy [157]. Researchers used the cis elements which were required to sustain the noncanonical splicing of the messenger RNA (mRNA) encoding XBP1 in response to ER stress, to induce the expression of heterologous genes producing IFN-α, resulting in a significant antiviral effect [157]. In addition, ER stress inhibitors such as 4-phenylbutyric acid (PBA) have been clinically used, although the purpose is not to treat viral hepatitis. In vitro, PBA showed its ability to block the production of HCV, suggesting itself a potential anti-HCV therapeutic option [158]. More studies proposed potential directions other than specific treatment options. The autophagic pathway is considered to be an important therapeutic target for liver disease, tightly intertwined with apoptosis [159]. CHOP plays an important role in both autophagy and apoptosis after the cell is infected. In studies on types of viruses other than hepatitis viruses, when the PERK-eIF2α pathway and the expression of CHOP are inhibited by small molecule inhibitors, the replication of the viruses will be limited [160]. If the importance of CHOP in the progression of viral hepatitis is confirmed in the future studies, inhibitors of the PERK-eIF2α pathway or CHOP or other modulators of UPR-autophagy pathways would be a direction for the treatment of viral hepatitis [161]. A recent study found that transcription of interleukin (IL)-8 can be induced by HBx and the large HBs protein via ER stress, indicating that IL-8 may be essential for the immune response to HBV and a potential target for anti-HBV therapy [130]. In HCV, autophagic flux impairment correlates with ER stress induced by HCV, making decreasing ER stress a promising therapeutic strategy for hepatitis caused by HCV [131].

## Figures and Tables

**Table 1:** Hepatitis B virus and associated ER stress pathways.

HBV Protein	Molecules	ER Stress Signaling Pathway	Mode of Action	References
HBsAg	PERK	PERK-eIF2α pathway	Activator of GRP78 and PERK; HCC development and recurrence	Li et al., 2019 [66]; Su et al., 2008 [86]
	IFN	UPR pathway	Inhibitor of UPR; Cell death promotion	Baudi et al., 2021 [87]
HBx	IRE1α	IRE1α-XBP1 pathway	Activator of IRE1α; Increase EDEM; Apoptosis inhibition	Li et al., 2007 [63]
	ATF6	ATF6 pathway	Activator of ATF6	Li et al., 2007 [63]
	SDF-1	PERK-eIF2α pathway, ATF6 pathway	Activator of UPR; Cell survival promotion	Cho et al., 2011 [11]; Cho et al., 2014 [88]
	COX-2	PERK-eIF2α-ATF4 pathway	Increase COX2	Cho et al., 2011 [11]
HBcAg	ATF6	ATF6 pathway	Activate ATF6 and mediate cell apoptosis	Nakanishi et al.,2005 [89]
	PERK	PERK-eIF2α pathway	Activator of GRP78 and PERK;	Choi et al., 2019 [90]
	IRE1α	IRE1α-XBP1 pathway	Activator of IRE1α; mediate cell apoptosis	Choi et al., 2019 [90]
	eIF2α	PERK-eIF2α pathway	Activator of eIF2α	Lee et al., 2015 [91]

Abbreviations: IRE1α, inositol-requiring enzyme 1α; HBx, hepatitis B virus X protein; XBP1, X-box-binding protein 1; EDEM, endoplasmic reticulum degradation-enhancing-mannosidase-like protein (EDEM); PERK, pancreatic endoplasmic reticulum kinase; eIF2α, eukaryotic translation initiation factor 2α; GRP, glucose-regulated protein; HCC, hepatocellular carcinoma; ATF, activating transcription factor; IFN, interferon; HBsAg, hepatitis B surface antigen; UPR, unfolded protein reaction; SDF, stromal cell-derived factor; COX, cyclooxygenase; HBcAg, hepatitis B core antigen.

**Table 2:** Hepatitis C virus and associated ER stress pathways.

HCV Protein	Molecules	ER Stress Signaling Pathway	Mode of Action	References
E2	PERK	PERK-eIF2α pathway	Inhibitor of PERK	Zhang et al., 2012 [58]
Core	CHOP	ATF6 pathways	HCV core-mediated apoptosis	Takahashi et al., 2017 [127] Benali-Furet et al., 2005 [128]
NS2	eIF2α	PERK-eIF2α pathway	eIF2α phosphorylation promotion	Bussche et al., 2010 [129]
NS3/4A	JNK	IRE1 pathway	Induce a mild apoptotic and oxidative stress response	Bussche et al., 2010 [129] Ríos-Ocampo et al., 2019 [130]
NS4B	XBP1, ATF6	IRE1α-XBP1 pathway, ATF6 pathway	Activator of IRE1α and ATF6EDEM ↓ ^1^	Zheng et al., 2005 [131] Tardif et al., 2004 [61]
NS5A	CHOP	PERK-eIF2α pathway	Increase the expression of CHOP	Meng et al., 2019 [132] Mishima et al., 2010 [133] Fang et al., 2013 [134]
E1	MHC Ⅰ	ATF6 pathway	Activator of ATF6	Tardif et al., 2003 [84] Selby et al.,1999 [135]

^1^ The expression was inhibited. Abbreviations: XBP1, X-box-binding protein 1; IRE1α, inositol-requiring enzyme 1α; EDEM, endoplasmic reticulum degradation-enhancing-mannosidase-like protein (EDEM); PERK, pancreatic endoplasmic reticulum kinase; eIF2α, eukaryotic translation initiation factor 2α; MHC, major histocompatibility complex; ATF, activating transcription factor; ER, endoplasmic reticulum.

## Data Availability

Not applicable.
